# Real Time Speed Estimation of Moving Vehicles from Side View Images from an Uncalibrated Video Camera

**DOI:** 10.3390/s100504805

**Published:** 2010-05-11

**Authors:** Sedat Doğan, Mahir Serhan Temiz, Sıtkı Külür

**Affiliations:** 1 Department of Geodesy and Photogrammetry, Engineering Faculty, Ondokuz Mayis University, 55 139 Kurupelit, Samsun, Turkey; E-Mail: mstemiz@omu.edu.tr; 2 Department of Geodesy and Photogrammetry, Civil Engineering Faculty, Istanbul Technical University, 80 626 Maslak, Istanbul, Turkey; E-Mail: skulur@srv.ins.itu.edu.tr

**Keywords:** vehicle speed estimation, video, traffic monitoring, optical flow

## Abstract

In order to estimate the speed of a moving vehicle with side view camera images, velocity vectors of a sufficient number of reference points identified on the vehicle must be found using frame images. This procedure involves two main steps. In the first step, a sufficient number of points from the vehicle is selected, and these points must be accurately tracked on at least two successive video frames. In the second step, by using the displacement vectors of the tracked points and passed time, the velocity vectors of those points are computed. Computed velocity vectors are defined in the video image coordinate system and displacement vectors are measured by the means of pixel units. Then the magnitudes of the computed vectors in image space should be transformed to the object space to find the absolute values of these magnitudes. This transformation requires an image to object space information in a mathematical sense that is achieved by means of the calibration and orientation parameters of the video frame images. This paper presents proposed solutions for the problems of using side view camera images mentioned here.

## 1. Introduction

In recent years, much research has been performed for developing real time traffic monitoring systems for managing the traffic flow of roadways, prevention of accidents, providing secure transportation, *etc.* Within these works, one aim is to realize different applications such as estimation of vehicle speeds on the roadways, determination of traffic intensity and if necessary, to direct the vehicles to less dense roads, manage the lighting times of traffic lights automatically, *etc.* [1–4]. But according to our literature survey, side view images hadn’t been used before for speed estimation in these applications except in [5]. The methods given in [5] use roadside line scan cameras for speed estimation and use different methods from ours.

In this paper, we present some of the first results of our ongoing research project on the problem of real time estimation of moving vehicles by using side view video images. To find vehicle speed, any digital video camera which acquires images in visible light spectrum may be used. Frame sampling rate, geometric and radiometric resolutions, and distortion amounts of the optical system of the camera affect the precision of the estimated speeds.

Solutions and the models to be used for speed estimation problem vary according to the applications and their final purposes. When applications related to vehicle speed estimation problems are investigated, two main fields are distinguished: traffic surveillance [6] and driver assistance systems or intelligent vehicle systems [7]. Traffic surveillance systems generally involve those applications which require global information on the general traffic situation of the roadways rather than individual vehicles travelling on the roads. For example, estimation of speed of traffic flow of a roadway at different times and dates [8,9], belongs to this group, as well as determination of the traffic density, timing of the traffic lights, signalisation works, *etc.* On the other hand, there are different applications which require speed information of each individual vehicle on traffic scenes. Furthermore, driver assistance systems and intelligent vehicle systems also require individual speeds of vehicles.

The starting point of many works for traffic surveillance applications are based on the segmentation of the moving objects, and for this purpose background subtraction methods are mostly used [3]. For this purpose, each pixel of the successive frame images are subtracted such that I(x,y,t) – I(x,y,t + Δt). The absolute value of this subtraction operation is used. In order to eliminate the object shadows, some other operations are often performed on the segmented images [10–13].

In this paper, we examine the problem of real time speed estimation of one moving vehicle from side view video images. The proposed solution to this problem may be used directly for traffic law enforcement to prevent the drivers from exceeding the speed limits. Furthermore, the proposed methods may also be used within a sensor network for active driver assistance and security systems. We are currently developing an intelligent sensor network to be used for both driver assistance and for automatic mobile vehicles [14]. Side view images and the proposed methods will be an important part of this network.

In order to solve the speed estimation problem of an individual vehicle using video frame images, many points which are identified on the image of the vehicle should be selected. Then, the displacement amounts of each selected point between two successive image frames and per unit time, should be found. Those displacement amounts per unit time are essentially equal to the instantaneous speeds of each point. These briefly explained tasks must be performed automatically and also within a very short time period of less than one second. Since the nature of the problem is ill posed, many technical problems relating to the above tasks must be solved. Even if we ignore the physical structure of the problem for a moment, the matters related to the selection process of the points to be tracked and tracking those selected points on the successive image frames involve difficult problems to be solved too. For example, because of the motion of the moving vehicle, if a selected point cannot be seen on the next frame or it falls into the out of vision range of the camera, what should be done? Some other problems are how will the time passed be measured? If displacement vectors of the points have been obtained in the image coordinate system, what will be their corresponding absolute values in the object space? It is possible to find solutions to those problems by using different approaches according to the underlying problem. In this paper, all of those problems mentioned above will be handled and we will give the first results of our ongoing studies on the proposed solutions to those problems. In conjunction with this, we will explain the approaches that we used to estimate the speed of a vehicle as well as the image processing procedure that we used to select the tracking points and computation of displacement vectors, *etc.*

## 2. Problem Statement, Methodology and Specifications

In this paper, we propose the real-time estimation of only one moving vehicle’s speed by using one video camera and side view images taken with it. Since it is not possible to extract 3D geometric information with one camera, in order to solve the speed estimation problem, some geometric constraints are required and the images should be taken under these constraints and the processing procedures should also be performed with those restrictions. For example, we assume that the imaged scene is flat. Perspective distortions on the acquired images must be either very small or of a degree that they can easily be rectified. Furthermore, by using only one camera, the velocity vectors can only be obtained in two dimensions, so the scale of the images along the 2D velocity vectors should be defined in a precise manner. For this purpose, at least the length of a line joining two points within the field of view of the camera and on the road and aligned along the velocity vectors, must be measured precisely. In this paper, we measured the lengths of two lines along the road by geodetic measurements using a simple measurement tape, within a precision of ±1 millimetre.

Since we define the scale of the images along the road, the field of view (FOV) of the camera must be set up so that it acquires the moving direction of the vehicles, *i.e.*, it must be set up so that it takes side view images of the vehicles. While this kind of acquisition plan provides advantages on the solution of the scale problem, on the other side it causes the imaging time of the vehicle to be shortened. In other words, entrance and exit time of a vehicle into the FOV of the camera is shortened and this situation also causes the time required for real time processing of all the procedures to be performed for speed estimation to be shortened too. A discussion on this disadvantageous situation is necessary. The precise specifications of the camera that we have used for the experiments in this paper are required for this discussion. We have used a camera with a frame rate of 30 fps and with an effective area of 640 × 480 pixel^2^. The pixel size which corresponds to the effective area of the camera is 9 microns. The focal length of the camera is 5.9 mm. We capture images in grey level mode at 30 fps (frames per second), meaning that a frame is captured within 33.3 milliseconds after the previous frame had been obtained, so in this case, all of the computations required for the speed estimation problem must also be completed within 33.3 milliseconds, which raises two very important questions at this time: (1) is it possible to perfom all of the computations within a very short time period (*i.e.*, in 33.3 milliseconds)? and (2) is it possible to track a point on the next frame, if the vehicle is moving too fast and the camera is very close to vehicle (namely to the road)?

In order to answer the first question, at first it should be noted that we use gradient based LK optical flow and we compute sparse optical flow rather than dense flow. This is a reasonable approach given the computation time of the each individual algorithms and their total computation time. Before giving this information, it will be better to give the flow of the overall process because this will enable the reader to see which algorithms have been used. In Table 1, the overall operations of our approach for speed estimation problem have been listed.

As seen in Table 1, the operations of step I are performed offline at the beginning of the speed estimation problem. After step I has been completed, the real time procedures begin. In Table 2, the computation times of each operation in step II have been given for a laptop configuration that had been used for real-time speed estimation experiments given in this paper. As seen in Table 2, the operations between 2.3–2.5 and 2.7–2.11 are very fast. Each of these operations is completed in microseconds and we could not measure the execution times in milliseconds (clock cycles). However, we can say that all of the operations are completed between 29–31 milliseconds. We have used OpenCV API functions to perform the operations 2.1–2.4. The rest of the operations 2.6–2.11 are performed with our own codes written with Borland C++ Builder 6.0 and pThread library. We have used multi threading computation with pThread library. The total time of the operations takes about 30 milliseconds for our real time applications with a laptop computer whose specifications have been given in Table 2. Here it should be noticed that the computation times are valid for the ROI region with the dimensions of 640 × 100 pixel^2^.

The total computation time of the whole image resolution, *i.e.*, 640 × 480 pixel^2^ changes between 35 × 125 milliseconds. Our routines are executed on the Windows XP platform. We have discarded all of the utility programs such as antivirus and other unnecessary Windows components. On the other side, to guarantee we can capture frames without any frame loss, our code controls the computation time while running in real-time mode. For example, if the computations end in 30 milliseconds, the program waits for 3.3 milliseconds for capturing the next frame. To capture a new frame, we force a capture command under the control of the program. In very rare cases, especially when some of the Windows programs are running, the computation time might increase. We have obtained the maximum delay time about 10 milliseconds when this situation occurs. *i.e.*, in this worst case, the computation time extends to 40 milliseconds. Although this case happens very rare, our program solves this problem in an adequate manner. When such a situation occurs, this causes two problems: one of them is the change of the time period between two frames which we use to find the speed, and the other one is mismatching risk of the corresponding points due to more much displacement of the points. The second risk increases especially when the vehicle is faster. In this case, if mismatching occurs, the number of erroneous velocity vectors increases. If there are enough number of correct matches and if the erroneous vectors had been eliminated, then the speed is computed together with those error free vectors and the passed time between frames. If the number of error free vectors is not enough, then these frame pairs are discarded and the procedure continues to work as usual. But we should note that when the Windows components and other utility programs are offline, we haven’t faced such a problem. On the other hand, it must be considered that a real time system executes with special embedded processors. *i.e.*, if the proposed methods are programmed for special processors, they will have better performance. Up to now, the first question has been answered. *i.e.*, our system can perform the necessary operations for speed estimation within very short time period (within 33.3 milliseconds).

Some comments should be given for the second problem too. Is it possible to track a point on the next frame, if the vehicle is too fast and the camera is very close to vehicle? There are two main factors which are to be considered for the speed estimation problem with one camera and with side view images taken with it. One of them is the Lucas Kanade algorithm (LK) that we used for tracking, and the other one is results from the physical nature of the problem. The physical problem is originated by the theory of relativity and from the image motion effect. This situation arises when both the camera is very close to vehicle and the vehicle is faster than the scanline sampling rate of the camera. This physical problem does not occur, if the limitations given in Table 3 are used, and so is out of the scope of this paper.

Now, let us explain the factors regarding the LK optical flow algorithm which is explained in detail in Section 6.2. The LK algorithm assumes that the displacement of a point is a few pixels (assumption 2 of Section 6.2). By using a pyramidal level approach, the algorithm can match the corresponding points, even if their displacements are substantially greater. When the level of the pyramid increases, the algorithm can match the more distant points. But in this case, the accuracy of the matching decreases [15]. According to our experiments, the algorithm can find the correct and accurate corresponding point if the displacement amount is about 90–150 pixels. However, we have set up the camera so that the maximum displacement of a point is about 50 pixels so the mismatching risk may be reduced and higher accuracy may be maintained. Now the matter is how to provide the maximum displacement constraint (*i.e.*, 50 pixels). The amount of displacement of a point depends on the scale of the images, frame rate of the camera and the speed of the vehicle. The scale factor of the images is related to the camera-to object distance and the focal length of the camera. Scale of a rectified image can be obtained approximately by the relation λ ≅ 1 + d/f, where λ is a scale factor, d is camera-to object distance and f is focal length of the camera. If a vehicle is moving with a velocity V km/h, its displacement amount per one millisecond is equal to 1/3600 × V m/ms (meters per millisecond) in object space. Object space displacement can be converted to image space by dividing it with the scale factor. This is the metric displacement amount of the vehicle in the image space. This metric unit can easily be converted to pixel units by using the dimensions of the effective image area and the pixel size of the camera. We assume that if the vehicle is moving with a speed which corresponds to a displacement amount smaller or equal to 50 pixels in the image space, our system can measure the speed of the vehicle correctly. In Table 3, we give some sample values which show the relations between camera-to-object distance and maximum vehicle speed that can be measured by our proposed method. The values in the table have been computed for the frame rate of 33.3 milliseconds and the focal length of the camera that we have used for our experiments given in this paper which is 5.9 mm.

In the following section, we explain the physical model of the speed estimation problem. In Section 4, rectification and the solution of the scale problem are given, in Section 5 selection of the tracking points and in Section 6, optical flow for the tracking of the selected points are explained with a real application sample from our test studies.

## 3. Physical Model of Speed Measurement by a Video Camera

In order to find the speed of a vehicle by a camera, we must find how the reference points, which are selected on the vehicle, change their positions in time. Since those points are stable on the vehicle (relative to vehicle itself) if the vehicle is moving relative to the observer (*i.e.*, relative to the camera) then those stable points must also move with the same speed and to the same direction as the vehicle is. In this work, since the observer camera is stable on the ground, the speed which is computed relative to the camera must be equal to the speed which is relative to the road which remains stable in front of the camera.

**Figure 1.**
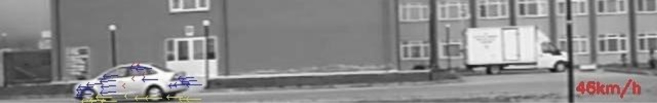
Velocity vectors before filtering of outliers.

To find the vehicle speed, successive frame images of the camera can be used. In this case, only the instantaneous speed can be found. This instantaneous speed is computed as follows:

(1) $$v=\frac{\Delta p}{\Delta t}$$

where **v** is instantaneous velocity vector of a point and **v ∈** R^2^ (*i.e.*, in 2D space since one camera is used), **Δp** is displacement vector of that point and **Δp ∈** R^2^. The displacement vector expresses the spatial displacement of a point during the time interval Δt. Here the time interval Δt is equal to the time which passes between two successive video frames and is equal to the frame replay rate (or frame capture rate) of the camera. In the experiments given in this paper, Δt is 33.3 milliseconds, which is the frame capture time of the camera that we used. As seen in Equation (1), we express the vectors with bold lowercase letters. Equation (1) gives the instantaneous speed (or velocity) of a point which is marked on the vehicle and selected for tracking. To find the velocity of the vehicle, only one point is not enough. During the selection of the points from the image of the vehicle, local approaches are used. If some errors occur during this selection step, the computed velocity vector will be affected by those errors and so the computed speed will be erroneous. For this reason, to estimate the speed of a vehicle, many more than one point should be selected and all of their instantaneous velocity vectors should be computed. Then by averaging the instantaneous velocity vectors of the whole selected points, the instantaneous velocity vector of the vehicle is found. For the formal expression, let us assume that n points are selected from the vehicle to be tracked and let **v_i_** (t) (i = 1, ..., n) represent the instantaneous velocity vectors of each of n points at time instance t. Then by using those instantaneous velocity vectors, we can find the instantaneous velocity vector of the vehicle by:

(2) $$v_{\text{iv}}(t)=\frac{1}{n}\sum_{i=1}^{n}v_{i}(t)$$

where **v_iv_** (t) is the instantaneous velocity vector of the vehicle at time instance t, **v_i_**(t) is the instantaneous velocity vector of i^th^ point on the vehicle and n is the number of the selected and tracked points. Here, it should be noted that, if some of the **v_i_** vectors are erroneous, then **v_iv_** will also be erroneous. So, before computing the instantaneous velocity **v_iv_** of the vehicle, the erroneous **v_i_** vectors must be eliminated. Then the value of n also changes, *i.e.*, number of the points decreases. For the elimination of the erroneous vectors, standard deviation of the n velocity samples can be used for fast evaluation. The instantaneous velocity of the vehicle should be computed by Equation (2), after the suspicious **v_i_** vectors had been eliminated. There are two very important error sources which affect the magnitudes of the velocity vectors in the significant level. One of them is the matching of the unwanted moving points on the background. The other one is the matched vehicle shadow points. We use an image subtraction method to eliminate the background and thus find the moving objects between the successive frames. All of the moving objects are visible on the difference image while stable objects are not. For example moving vehicle and its shadow, oscillating grasses, waving trees, *etc.* are all visible in the difference image and are candidate features to be tracked. Changing illumination conditions might also be detected as a candidate moving object. We use a histogram thresholding algorithm to eliminate such unwanted background oscillations and illumination effects. We further eliminate the shadow points. But although the use of the thresholding algorithm, some of the erroneous (unwanted) points may still be reside and those points may be tracked as if they were vehicle points. It is clear that those unwanted points must move with the velocities which are significantly different from the vehicle points. If those points are not filtered, the computed vehicle speed will be erroneous. In Figure 1, both erroneous and error free velocity vectors have been shown in different colours. The image shows the ROI region that was used for real time operations in our system.

In Figure 1, the velocity vectors with the blue colour are error free correct vectors, yellow shows the velocity vectors of the shadow points and the red shows the mismatched point vectors. The image in the figure has been taken from a real experiment that we performed with our proposed system. The displacement amounts of all points (magnitudes of the vectors), which have been shown in the Figure 1 have been given in Table 4. The units of the displacements are in pixels.

**Figure 2.**
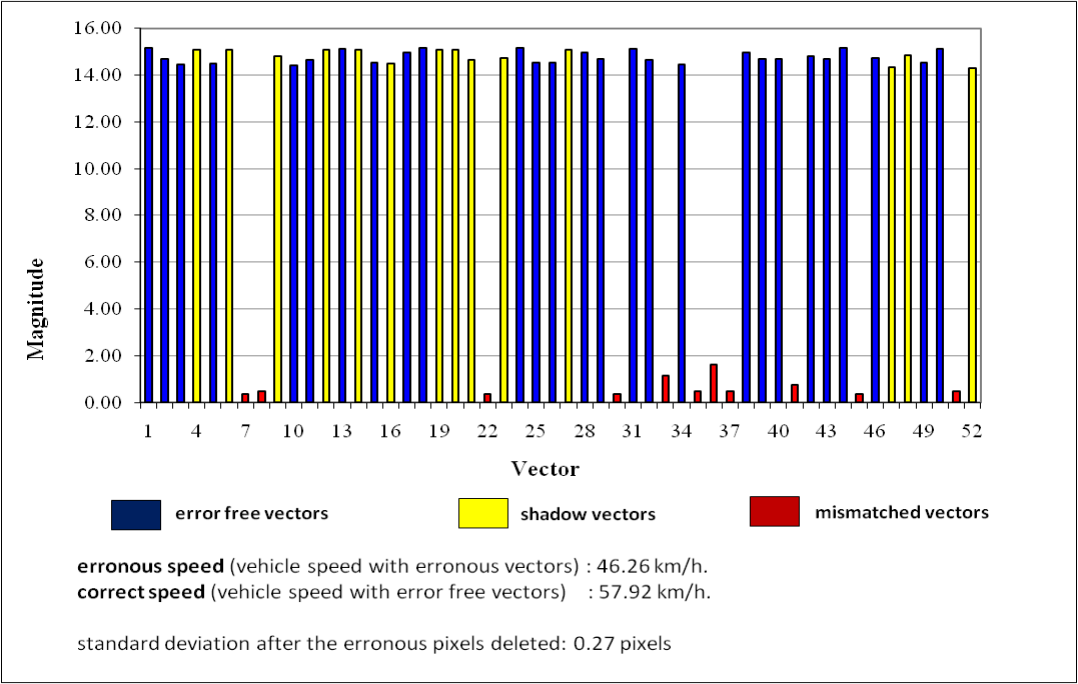
Graphical representation of vectors.

As seen in Table 4, some of the magnitude values are much smaller than others. These vectors are probably erroneous. We assume that if ||*v*|| ≤ ||*Mean – Standard deviation*||, the velocity vector **v** is erroneous. Now here there is an interesting situation. To see this, it is better way to give the graphical representation of the velocity vectors which are given in Table 4. Magnitudes of the shadow vectors are very close to the magnitudes of the error free vectors, as seen in Figure 2. This means that the above standard deviation threshold does not eliminate the shadow vectors in this experiment. But however, the shadow vectors seem to have same speed as the error free vehicle vectors. This situation can also be observed in Figure 1. The shadow of the vehicle is moving with almost the same speed as the vehicle. But this is a lucky strike. Because, the incidence angle of the sunlight (azimuth angle) was almost normal to the earth surface during our experiment. If this incidence angle changes within a day, the length of the shadow also changes as the vehicle is moving. In this case, the shadow speed will change randomly and independent from the vehicle speed. The changes which exceed the threshold value of our assumption will be eliminated. But otherwise will not be eliminated and then they will be closer to the vehicle speed. In this case, even if the undeleted shadow vectors are used, the speed of the vehicle can be obtained in an adequate approximation. But when there are artificial light sources such as street lamps in the evening times, then the shadows should be eliminated. So in all cases, we eliminate the shadow vectors with an explicit elimination procedure.

In Figure 2, there are 11 mismatched, 14 shadow and 27 error free vectors. This means that 52 points have been matched between previous and current frames but only the 27 of them are correct and should be used for speed estimation.

**Figure 3.**
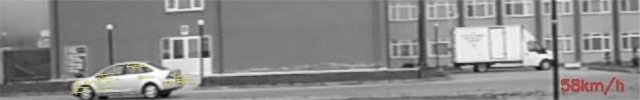
Error free vectors and speed.

In Figure 3, a screen shoot of our system with the error free vectors and the correct speed have been illustrated.

When the vehicle enters the FOV of the camera for the first time, the instantaneous speeds of the vehicle at time instances between each successive image frames, should be computed continuously until it leaves the FOV. By using those computed instantaneous speeds, the average speed of the vehicle, during the time interval that passed between the entrance and exit times, can be found. Let I(t_1_), …, I(t_m_) be the frame images of the vehicle at time instances t_j_ and m is the number of frames on which the vehicle is apparent. Then by using the instantaneous speeds v_iv_(t_j_) of the vehicle which are computed at time instances t_j_ between the frames I(t_j_) and I(t_j + 1_) where (j = 1, …, m), average speed of the vehicle can be computed as:

(3) $$v_{\text{avg}}=\frac{1}{m}\sum_{j=1}^{n}v_{\mathrm{iv}}\left(t_{j}\right)$$

where **v**_avg_ is the average velocity of the vehicle, **v_iv_**(t_j_) is the instantaneous speed of the vehicle at time instance t_j_ and m is the number of instances namely the number of image frames on which the vehicle is apparent. Three difficult problems should be solved to find the speed of a vehicle by the explained physical approach above. Those are: (1) solution of the scale problem to find the absolute speed of the vehicle, (2) selection of the points to be tracked from the image of the vehicle and (3) tracking of selected points and computing the velocity vectors. In the following chapter the first problem and in the Section 5 the next problem and their solution methods are discussed.

## 4. Rectification of Frame Images with Vanishing Points

**Figure 4.**
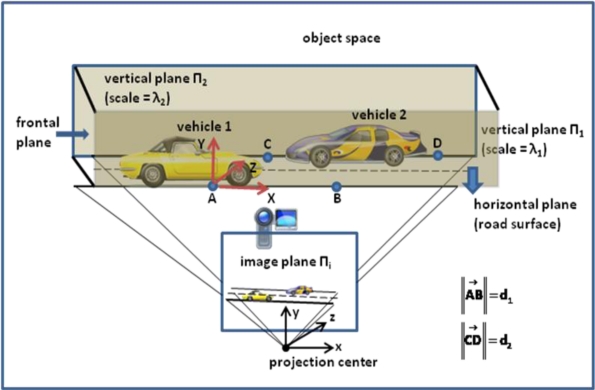
Sideview image acquisition plan.

In order to find absolute values of displacement vectors or velocity vectors in object space, the vectors computed in video image coordinate system should be transformed to the object coordinate system which is in the object space. For this purpose, we assumed some restrictions as explained in Section 2. For example, we assumed that the observed scene is flat. But on the other hand, we acquire sideview images of the vehicle as seen in Figure 4. In this case, our flatness assumption is made for the visible side of the vehicle. In ideal situation, the flat scene must be just as vertical planes as in the figure. The distances from the camera to vertical planes are different because of the different depths. This difference causes the planes to have different scales in the image plane. On the other side, with only one camera and one image, it is not possible to detect the depths and indirectly the scales of the planes on the image.

In order to solve this scale problem, we simply measure two distances in the object space with a measurement tape. These measured distances are lying on the two vertical planes and along the borders of the road. The vehicle travels on the road surface, either from left to right or from right to left. These moving directions are shown with vehicle 1 and vehicle 2 respectively, in the Figure 4. With this configuration, only one side of the vehicles is visible and these visible sides must be parallel to the vertical planes on the image in ideal situation. This ideal situation is achieved when the image plane is parallel to the vertical planes. In this ideal situation, what can be said about the scale factor?

In order to answer this question, first let’s assume that the vehicle is moving from left to right as vehicle 1 as in Figure 4. Then the visible side of the vehicle is right side and it is closer to plane Π_1_ with the scale λ_1_. Scales of the vertical planes Π_1_ and Π_2_ are obtained with the measured distances d_1_, d_2_ and their corresponding distances d′_1_ and d′_2_ on the image plane such that λ_1_ = d_1_’/d_1_ and λ_2_ = d_2_’/d_2_ respectively. In s similar way let’s assume that the vehicle is moving from right to left. Then its visible side is the left side and it is closer to centre of the road axis. In this case, the scale can be taken as λ = (λ_1_ + λ_2_)/2. According to this configuration and assumptions, if the ideal situation is achieved, then absolute values of the velocity vectors or displacement vectors can be obtained by using the corresponding scale factors.

**Figure 5.**
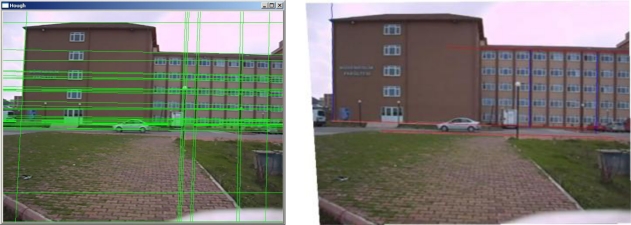
Vanishing lines found with Hough transformation (left) and rectified image (right).

If the image plane is in the ideal case, then any parallel line in the vertical planes must remain parallel in the image plane. Similarly, the parallel lines on the horizontal plane must also remain parallel in the image plane. If the image plane is far away from the ideal situation, these parallel lines will not be parallel in the image plane. This means that those parallel lines in the object space intersect each other in the image plane. Intersection points of the parallel lines are known as vanishing points. By using vanishing points and their corresponding vanishing planes at the horizontal and vertical directions, the images can be rectified by using vanishing points geometry [16–19], so that they represent the ideal case. For this purpose, we first find vanishing lines by using the Hough transformation and by computing their intersection points we obtain the coordinates of the vanishing points in the image coordinate system. By using those vanishing points we rectify the image by making the vanishing lines parallel to each other. In Figure 5, vanishing lines computed by the Hough transformation are given from our sample application.

In our acquisition plan, the camera is stable. In this case, when the rectification parameters are found for the first time, they can be used until the camera changes its position. Thus, at the beginning of the speed estimation application, at first the rectification parameters can be found for the first time and these parameters can be used as long as the camera stays stable.

For the speed estimation problem, after rectification parameters have been found, it is not necessary to rectify the whole image. Instead, only the selected and tracked point coordinates may be rectified for speed improvement of the real time computational cost. But however, we give the wholly rectified image on the right image of Figure 5, for visual evaluation of the reader. After the rectification step, by using the rectified velocity vectors (or scale corrected velocity vectors) the absolute speed of the vehicle is found.

## 5. Automatic Selection of Points to be Tracked from the Images of the Vehicle

In order to track moving objects with video images, points to be tracked which belong to the object on the successive video frames, should be selected automatically. It is well known that good features to be tracked are corner points which have large spatial gradients in two orthogonal directions. Since the corner points cannot be on an edge (except endpoints), aperture problem does not occur. One of the most frequently used definitions of a corner point is given in [20]. This definition defines a corner point by a matrix which is expressed by second order derivatives. These derivatives are partial derivatives of pixel intensities on an image and are ∂^2^x, ∂^2^y and ∂x∂y. By computing second order derivatives of pixels of an image, a new image can be formed. This new image is called “Hessian image”. The name “Hessian” arises from the Hessian matrix that is computed around a point [21]. The Hessian matrix in 2D space is defined by:

(4) $$\left[\begin{array}{ll} \frac{\partial^{2}I}{{\partial x}^{2}} & \frac{\partial^{2}I}{\partial x\partial y}\\ \frac{\partial^{2}I}{\partial y\partial x} & \frac{\partial^{2}I}{{\partial y}^{2}} \end{array}\right]$$

Shi and Tomasi in [22] suggest that a reasonable criterion for feature selection is for the minimum eigenvalue of the spatial gradient matrix to be no less than some predefined threshold. This ensures that the matrix is well conditioned and above the noise level of the image so that its inverse does not unreasonably amplify possible noise in a certain critical directions.

When it is desired to extract precise geometric information from the images, the corner points should be found within a subpixel accuracy. For this purpose, the all candidate pixels around the corner point can be used. By using the smallest eigenvalues at those points, a parabola can be fitted to represent the spatial location of the corner point. The coordinates of the maximum of the parabola is assumed to be the best location for being a corner. Thus the computed coordinates are obtained in subpixel precision. For the subpixel selection methods readers are referred to [23].

In our system, as soon as the camera begins for image acquisition, points are selected continuously in real time from the frame images. On the first frame, points are selected and on the next frames those points are tracked and instantaneous velocity vectors of those points are computed. In our system, we restrict the number of points between 20 and 500 and at least 20 points are selected in the worst case.

## 6. Tracking of Selected Points and Estimation of Speed

For speed estimation, correspondence of each selected point on the first frame on which the vehicle appears for the first time, must be found on the next (successive) frame. In the ideal case, correspondence of a selected point must be the same point on the next frame. In order to find the corresponding point, there is no prior information other than the point itself, and it seems that it is not also possible to find exactly where the match point is. Since only one camera is used, it is not possible to define a search area on the next frame which will restrict the search space with some geometric constraints such as epipolar geometry. From this it can be said that, it is impossible to use stereo matching approaches. Instead, if we assume that each image in the each frame is flowing by the very short time period and thus changing the position during the flow, then a modelling approach which models this flow event can be used. These kinds of flow models are called “optical flow”. First we will briefly explain the optical flow approach, and later explain the methods that we used in this paper.

### 6.1. Optical Flow

**Figure 6.**
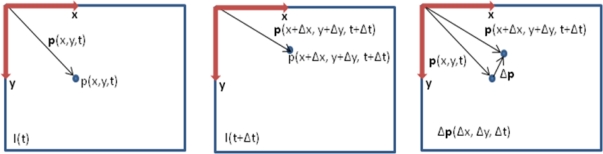
Optical flow.

Let p(x,y) be a corner point on a 2D image space, where (x,y) are the image coordinates of the point p. I(t) be a video frame image which was taken in time instance t. Then a point on that frame, namely p € I(t), may be expressed by its position vector as **p**(x,y,t). According to this formal expression, all points p_i_(x_i_, y_i_, t) € I(t) can be expressed by position vectors whose starting points are on the origin of the image coordinate system and their end points are equal to points p_i_s as in Figure 6.

As can easily be seen in Figure 6, instantaneous velocity vectors of the points are vectors obtained by scaling the **Δp** displacement vectors with passed time Δt. Since **Δp** displacements are two dimensional, velocity vectors are two dimensional too. In other words, a velocity vector **v** has two components (v_x_,v_y_). From the above explanation we see that, after two successive frames had been processed, each tracked point is assigned a velocity vector or equivalently a displacement vector. If this point-velocity vector assignment operation is performed for not only the selected points but also the whole pixels on the image, this is called “dense optical flow”. In this case, velocity vector field of the whole image can be obtained. The Horn and Schunk method given in [24] is one of the first examples based on the dense optical flow which finds the velocity vector field of the whole image. But there are vast amount of pixels in an image and velocity vector computation of such a vast amount of points is computationally very expensive. So, dense optical flow methods are less appropriate for the real time applications unless embedding programmable chips are used. Instead of dense optical flow, velocity vectors may also be computed for only the selected points which are not dense points. Thus, a sparse velocity vectors can be obtained. Such a method, which computes the optical flow with less amount and sparse points is called “sparse optical flow”. In this paper, since we use only the selected corner points for the speed estimation problem, we preferred to use sparse optical flow method.

### 6.2. The Lukas-Kanade (LK) Optical Flow Method

When only one video camera is used, there is no information other than themselves of the selected points to find their correspondences on the next frame. For this reason, it is not possible to know exactly where the corresponding points are on the next frame. But however, by investigating the nature of the problem, some assumptions may be made about the possible locations where the corresponding points might be located. In order to ensure these assumptions are as close to the physical reality as possible, there must exist a theoretic substratum at which these assumptions are supported. Furthermore, this theoretic substratum must be acceptable under some certain situations.

In this sense, it is first necessary to decide and define what information is to be used for finding correspondences. According to this reasoning, the first thing evoked in the mind is the idea of looking at the texture, colour or intensity of the neighbouring area of a selected point in the first frame and expecting that its correspondence on the next frame must also has the same or nearly the same structural properties by the means of texture, intensity or colour, *etc.* If this idea and related expectation really occur in the fact, then those texture and intensity information may be used for solution of the correspondence problem. Of course, only this information is not enough alone for the solution of the problem. Because, there may exist many candidate points which may have the same texture and intensity characteristics. In this case, a unique solution cannot be guaranteed. If so, there must be another assumption(s) which will help to guess the correct geometric location of the corresponding point. Lukas and Kanade have cleverly given three assumptions for the solution of the correspondence problem in their paper [25]. The assumptions of Lukas-Kanade Method are explained in the following paragraphs:

***Assumption 1:*** *Intensity values are unchanged*. This assumption asserts that the intensity values of a selected point **p**(t) and its neighbours on the frame image I(t), do not change on the next frame I(t + Δt), where Δt is too short time period less than one second. When the time interval Δt that passed between two successive image frames is too short, it can be seen that really the possibility of the occurrence of this assumption is too high. Because, in a very short time period which is measured in milliseconds, the effects such as the lighting conditions of the scene medium *etc.* that cause the intensity values to be changed must not lead to meaningful change effects since the time is too short. Of course, it is not possible that the assumption is 100% correct. For this, the vehicle should be stopping and the sampling rate of the video camera should infinitely be small. But however, within a very short time that is about 30 milliseconds, even if the vehicle is moving with a speed about 250 km/h, the possibility of the assumption to be real is still very high. But, between the image frames I(t) and I(t + 2Δt), anymore it is very low possibility that the assumption would be real.

***Assumption 2:*** *Location of a point between two successive frames changes by only a few pixels*. The reasoning which the assumption is based on, is similar to the reasoning of the first assumption. Between the frame images I(t) and I(t + Δt), when Δt is getting smaller, then the displacement amount of the point also gets smaller. According to this observation, a point **p**(t) at (x,y) coordinates of image I(t) will be at the coordinates (x + Δx, y + Δy) on image I(t + Δt) and these new coordinates will be closer to the previous coordinates within a few pixels. Thus the positions of the corresponding points on both images will be very close to each other. If this assumption is verified to be valid, then it is mentally a good approach to search the correspondence of a point that is on the first frame, around a region closer to its coordinates (*i.e.*, in the neighbouring region) on the next frame. Of course, it is not expected that this assumption to be 100% correct, as in the case of the previous assumption.

***Assumption 3:*** *A point behaves together with its neighbours*. The first two assumptions, which are assumed to be valid for a point must also be valid for its neighbours. Furthermore, if that point is moving with a velocity **v**, then its neighbours must also move with the same velocity **v**, since the motion duration Δt is too short.

The three assumptions above help develop an effective target tracking algorithm. In order to track the points and to compute their speeds by using the above assumptions, it is necessary to express those assumptions with mathematical formalisms and then velocity equations must be extracted by using these formalisms. For this purpose, if the symbolic expressions given in Figure 6 are used, the first assumption can be written in mathematical form as follows:

(5) I(p(x,y,t),t)=I(p(x+Δx,y+Δy,t+Δt),t+Δt)

where I(**p**,t) is the intensity value of a point **p** on the image I(t) which was taken at the time instant t. Note that the geometric location of the point is expressed by its position vector **p** € R^2^ (*i.e.*, in 2D space). Here I(**p**,t) expresses the intensity value of a pixel at the point **p** on the frame image I(t). In similar way, the right side of the equation expresses the intensity value of the corresponding pixel at the point **p +** Δ**p** on the frame image I(t + Δt). Accordingly, Equation (5) says that the intensity value of the point **p** on the current image frame does not change during the time period Δt that passed. In other words, it expresses that the intensity I(**p**,t) does not change by the time Δt. In the more mathematical sense, change rate of I(**p**,t) iz zero over the time period Δt. This last situation is formally written as follows:

(6) $$\frac{\partial I(p(x,y,t),t)}{\partial t}=0$$

If the derivative given in Equation (6) is computed by using the chain rule of derivative, we obtain:

(7) $$\frac{\partial I(p(x,y,t),t)}{\partial t}=\frac{\partial I(p,t)}{\partial p}\frac{\partial p(t)}{\partial t}+\frac{\partial I}{\partial t}=0$$

In Equation (7), the derivative ∂*I*/∂***p*** is spatial derivative at point **p** on the image frame I(t). Thus it can be expressed by ∂*I*/∂***p*** = **▿***I*. We can write this expression in explicit form as follows:

(8) $$\nabla I=\frac{\partial I}{\partial x}i+\frac{\partial I}{\partial y}j=I_{x}i+I_{y}j$$

The derivative ∂***p***(*t*)/∂*t* can also be written in a more explicit form:

(9) $$\frac{\partial p(t)}{\partial t}=\frac{\partial }{\partial t}(xi+yj)=\frac{\partial x}{\partial t}i+\frac{\partial y}{\partial t}j$$

If Equation (9) is investigated carefully, it can easily be seen that the vector (∂*x*/∂*t*) ***i*** is equal to the velocity of the point **p** in the x-axis direction. In other words, it is the x component namely v_x_ component of the velocity vector **v**. In similar way, the vector (∂*y*/∂*t*) ***j*** is the y component namely v_y_ component of the velocity vector **v**. Now we can rewrite the Equation (9) as follows:

(10) $$\frac{\partial p(t)}{\partial t}=v=v_{x}i+v_{y}j$$

If again Equation (7) is investigated, it is seen that the derivative ∂*I*/∂*t* = *I_t_* expresses the change rate of the intensity values at point **p**, between the frame images I(t) and I(t + Δt). Thus, Equation (7) can be rewritten as follows:

(11) $$\nabla Iv+I_{t}=0$$

where:

(12) $$\nabla I=\left[\begin{array}{l} I_{x}\\ I_{y} \end{array}\right]\;\text{and}\;v=\left[\begin{array}{l} v_{x}\\ v_{y} \end{array}\right]$$

Then Equation (11) can be written as:

(13) $$\left[I_{x}\;\;\;\;I_{y}\right]\left[\begin{array}{l} v_{x}\\ v_{y} \end{array}\right]=-I_{t}$$

The values of I_x_, I_y_ and I_t_ in Equation (13) can easily be computed from the frame images. The variables v_x_ and v_y_ are two unknown components of the velocity vector **v** and these are respectively the components in the directions x and y axes of the image coordinate system. In Equation (13), we have two unknowns to be solved, but we only have one equation. Since only one equation is not enough for unique solution of the unknowns, at the moment it seems not possible to solve these unknowns. In order to solve these two unknowns, we need more independent equations. For this purpose, the third assumption of the LK algorithm is used. That is, point **p** behaves together with its neighbours. So its neighbours must also satisfy the Equation (13). In other words, neighbour points (or pixels) of point **p** must move with the same velocity **v**(v_x_,v_y_). According to these explanations, the same equations as (13) are written for 3 × 3 or 5 × 5 neighbourhood of the point **p**. In this case, we totally have 9 or 25 equations with the same unknowns v_x_ and v_y_. Now the unknowns can be solved with overdetermined set of Equations (13) by using least squares or total least squares estimation method.

During the real time tracking, some selected points may not be seen on the next frame. This situation may arise because of different reasons. Especially, when the vehicle is entering into or exiting from the FOV of the camera, the possibility of occurrence of this situation is too high. In order to prevent such situations, we have interpreted the algorithm with the image pyramid approach, which uses coarse to fine image scale levels. For details of the image pyramid approaches, we refer the reader to [15] and [21]. In this case, in the coarse levels of the pyramid we use Equation (1) to find the velocity vector of each point by finding the displacement vector Δ**p** of each point by using matched coordinates and in the finer level of the pyramid we find the velocity vectors precisely by using Equation (13). We use Equation (2) to compute instantaneous speeds of the vehicle, and to compute the average speed of the vehicle we use Equation (3) by substituting the computed point velocity vectors.

Accuracy of the estimated speed of our system is ±1.12 km/h. We tested the system by comparing the estimated speeds to GPS measured speeds. One another way of the accuracy test is comparison of the estimated speeds to the measurement results of a speed gun, as described in [26]. But we could not find a speed gun, so instead of it we have used a GPS receiver (Magellan Triton 400), which measures speeds with very high accuracy about 0.1 knot (0.05 m/s) or 0.018 km/h [27,28]. For testing purposes we have connected the GPS receiver to another laptop computer just as described in [27] and [28]. The GPS receiver and another laptop computer which has been connected to receiver have been located in the vehicle during the travel. One operator has operated the GPS measurement system. When the vehicle enters into the FOV of the camera, laptop begins to record the speeds measured by the GPS. To record the GPS measurements in real time, we have coded a simple C++ function. Before the test measurements, we have marked the entrance and exit points on the road at which the FOV scene begins and ends. When the vehicle comes to the entrance flag, the operator starts the GPS and the laptop begins to record the measured speeds successively. When the exit flag is reached, the operator stops the recording procedure. At the same time, our system estimates and records its own estimated speeds. In Table 5, some of the test measurements are shown.

We can assume that the GPS speed measurements are accurate and error free because its accuracy is very high, as given by [27] and [28]. Then the relative errors of our estimation method are obtained by computing the difference between GPS and proposed method. By using the values given in Table 5, a mean squared error of the proposed method of about ±1.12 km/h is obtained.

## 7. Conclusions

In this paper, we have explained the real time speed estimation problem and its solution for one vehicle by using side view video images of the vehicle. The accuracy of the estimated speed had been obtained and is approximately ±1.12 km/h. The sparse optical flow technique is a very effective technique for the speed estimation of the vehicles. When considering more than one vehicle, speeds of each vehicle can also be found at the same time with the proposed methods. However in this case, estimated velocity vectors of the tracked points should be classified to find which vector belongs to which vehicle on the scene. For classification purposes, a classification scheme such as clustering methods may be used. For the speed estimation of multiple vehicles on the same scene, different methods than our proposed approach may also be used. For example, before selection of the points to be tracked, each vehicle can be segmented by using a parametric or geometric active contour and then the deformation of those contours may be tracked to find the speed of each vehicle separately. But this approach is not very suitable for real time speed estimation due to its computational costs. Our proposed method may also be used for automatic driver assistance systems if it is used within a real time sensor network. We continue to work on estimation of the speed of vehicles by cameras mounted on a moving vehicle and both using side view, front view and rear view images of the moving vehicles within a real time local network architecture.

## Figures and Tables

**Table 1.** Overall operations of proposed speed estimation process.

Step I Operations (performed offline)	Step II Operations (real time operations )

1.1. Capture frame I and Frame II 1.2. Compute the rectification parameters with vanishing point geometry 1.3. Store the rectification parameters 1.4. Enter the distance measurements for scale computation 1.5. Define a ROI region where the road and vehicle are visible	2.1. Capture frame i 2.2. Capture frame i + 1 2.3. Find difference ROI image 2.4. Eliminate background changes with histogram thresholding. 2.5. Select tracking points from the foreground (vehicle) image 2.6. Find corresponding points 2.7. Rectify the coordinates of the selected and the tracked points 2.8. Compute velocity vectors 2.9. Compute mean and standard deviations of the vectors 2.10. Eliminate outlier vectors 2.11. Compute the average instantaneous speed of the vehicle 2.12. Go to 2.2

**Table 2.** Computation times of real time operations.

**Real time operation**	Computation time (in milliseconds)*	**Explanation**
2.3. Find difference ROI image	< 1.0	completed in microseconds
2.4. Eliminate background changes with histogram thresholding.	< 1.0	
2.5. Select tracking points from the foreground (vehicle) image	10–12	
2.6. Find corresponding points	14–16	
2.7. Rectify the coordinates of the selected and the tracked points	< 1.0	completed in microseconds
2.8. Compute the velocity vectors	< 1.0	
2.9. Compute mean and standard deviations of the vectors	< 1.0	
2.10. Eliminate outlier vectors	< 1.0	
2.11. Compute the average instantaneous speed of the vehicle	< 1.0	
Total execution time	29–31	

* Laptop configuration: Intel core 2 Duo CPU, 2.40 GHz, 2 GB RAM

**Table 3.** Camera to object distance and maximum speed that can be measured.

**Focal length: 5.9 mm. Fps: 30**	**Distance (m)**	**Max speed (km/h)**	**Explanation**

	10	75	
	22.95	171	used in this paper
	26.20	196	
	30	224	
	40	300	

**Table 4.** Magnitudes of all vectors in pixels.

**Vector**	**Magnitude**	**Vector**	**Magnitude**	**Vector**	**Magnitude**	**Vector**	**Magnitude**

**1**	15.17244	**14**	15.09534	**27**	15.10201	**40**	14.67062
**2**	14.67051	**15**	14.53567	**28**	14.97215	**41**	0.75555
**3**	14.44615	**16**	14.48191	**29**	14.67011	**42**	14.79012
**4**	15.09515	**17**	14.97209	**30**	0.37625	**43**	14.67086
**5**	14.48138	**18**	15.17195	**31**	15.12538	**44**	15.17658
**6**	15.10202	**19**	15.09523	**32**	14.63253	**45**	0.36652
**7**	0.367685	**20**	15.09504	**33**	1.14171	**46**	14.73801
**8**	0.478954	**21**	14.64967	**34**	14.44434	**47**	14.34300
**9**	14.81166	**22**	0.37704	**35**	0.47859	**48**	14.84108
**10**	14.42731	**23**	14.73827	**36**	1.63186	**49**	14.52454
**11**	14.63479	**24**	15.17197	**37**	0.47909	**50**	15.11971
**12**	15.09527	**25**	14.52401	**38**	14.97558	**51**	0.47341
**13**	15.11739	**26**	14.52534	**39**	14.67038	**52**	14.29117

**Mean: 11.80376 pixels**

**Standard deviation: ±5.8522483 pixels, Absolute average instantaneous velocity of the vehicle: 46.26 km/h. (erroneous speed)**

**Table 5.** Accuracy test measurements.

**Experiment #**	**Vehicle Direction** LR: left to right RL: right to left	**Estimated Speed** (km/h)	**GPS Speed** (km/h)	**Difference** (errors relative to GPS measurements) (km/h)

1	LR	38.26	38.6	0.34
2	RL	36.73	38.5	1.77
3	LR	37.41	38.5	1.09
4	LR	47.61	48.3	0.69
5	RL	57.92	57.7	−0.22
6	LR	57.50	57.0	−0.50
7	RL	64.25	63.2	−1.05
8	LR	68.92	67.3	−1.62
9	RL	75.35	76.9	1.55
